# Repetitive Transcranial Magnetic Stimulation for Generalized Anxiety Disorder: A Systematic Literature Review and Meta-Analysis

**DOI:** 10.1093/ijnp/pyab077

**Published:** 2021-11-14

**Authors:** Tapan K Parikh, Jeffrey R Strawn, John T Walkup, Paul E Croarkin

**Affiliations:** 1 Pritzker Department of Psychiatry and Behavioral Health, Ann and Robert H. Lurie Children’s Hospital of Chicago, Chicago, Illinois, USA; 2 Northwestern University Feinberg School of Medicine, Chicago, Illinois, USA; 3 Anxiety Disorders Research Program, Department of Psychiatry and Behavioral Neuroscience, University of Cincinnati, Cincinnati, Ohio, USA; 4 Mayo Clinic, Rochester, Minnesota, USA

**Keywords:** Anxiolytic, clinical trial, Hamilton anxiety rating scale, neuromodulation, TMS

## Abstract

**Background:**

Anxiety disorders such as generalized anxiety disorder (GAD) impact 10% of the US population, and many patients do not completely respond to first-line treatments (e.g., selective serotonin reuptake inhibitors, serotonin-norepinephrine reuptake inhibitors, and psychotherapy). Given the dearth of evidence for non-pharmacologic, non-psychotherapeutic interventions, we performed a systematic review and meta-analysis of repetitive transcranial magnetic stimulation (rTMS) in adults with GAD.

**Methods:**

A systematic literature review using the Preferred Reporting Items for Systematic reviews and Meta-Analyses guidelines was conducted. Pre- and post-treatment anxiety scores were extracted, and a random-effects meta-analysis was conducted to determine the magnitude of improvement (standardized mean difference). Standard assessments of heterogeneity (e.g., Q-statistic, I^2^, and τ ^2^) and publication bias were performed.

**Results:**

The initial search resulted in 3194 citations, of which 6 studies were included in the meta-analysis. In total, 152 patients were studied, including 97 patients who received active treatment and 55 who received sham treatment, and heterogeneity was modest (I^2^ 13.32, Q = 5.77). In patients with GAD, rTMS produced a standardized mean difference of −1.857 (confidence interval: −2.219 to −1.494; *P* < .001) with a prediction interval of −2.55 to −1.16.

**Conclusions:**

The results suggest a robust effect of rTMS in GAD in the context of limited, heterogenous studies. Rigorously designed, randomized controlled trials of rTMS for GAD and related anxiety disorders are urgently needed. These studies will provide opportunities for biomarker development and integration of concurrent evidence-based psychotherapy to maximize results.

One in 10 adults meet DSM-5 criteria for an anxiety disorder in any given year (American Psychiatric Association, 2013). Anxiety disorders often emerge in childhood and are associated with substantial disability. Generalized anxiety disorder (GAD) is one of the most common anxiety disorders, characterized by a chronic, recurrent course (Strawn et al., 2021). In the Unite States, GAD accounts for 110 million disability days annually and may confer the greatest economic burden related to disability among all anxiety disorders (Revicki et al., 2012). Pharmacologic treatment of GAD often involves selective serotonin reuptake inhibitors and serotonin-norepinephrine reuptake inhibitors. Importantly, 50% of patients may not respond to the first-line treatment (Ansara, 2020), and treatment resistance in GAD is understudied. There are limited data for the second-line or augmentation strategies. Given common, inadequate treatment outcomes and shortcomings of available treatments (e.g., selective serotonin reuptake inhibitors, serotonin-norepinephrine reuptake inhibitors, combined use of medications and psychotherapy), novel, brain-based, targeted interventions are urgently needed. One such treatment, repetitive transcranial magnetic stimulation (rTMS or TMS), was first approved by the US Food and Drug Administration for major depressive disorder treatment in 2008. Treatment with TMS may have utility in anxiety disorders (Lisanby et al., 2002).

The initial studies and conceptual models regarding the application of TMS for GAD are promising. However, there are substantial knowledge gaps with how and when to dose TMS for anxiety disorders in adults and youth. Systematic information would assist with hypothesis generation, conceptual models, and pivotal study designs. This study sought to perform an exhaustive systematic review and meta-analysis focused on treating GAD with TMS. It was expected that existing TMS studies of anxiety would demonstrate comparable effect sizes with depression trials of TMS. A systematic review was conducted in accordance with the Preferred Reporting Items for Systematic reviews and Meta-Analyses guidelines. A medical librarian assisted with the development and execution of a suitable search strategy. Overall, 5367 citations were identified by the searches; after deleting the duplicates, this number was 3194, of which 202 potentially eligible articles were retrieved. Thereafter, 59 were selected based on therapeutic TMS use in anxiety disorders. The final inclusion step involved selecting articles that studied GAD as a primary or co-morbid diagnosis and studied the role of TMS in anxiety disorders, whether done in a randomized controlled trial or as an open-label trial. Case reports and case series were excluded. Six studies were finally included for the meta-analysis.

For each study, the number of patients included in the study were collected along with the rating scales used to assess the differences in anxiety rating pre- and post-treatment. These data primarily included an anxiety rating scale that was performed and reported as a mean score SD as well as the same numbers post-treatment. For example, if anxiety was assessed using Hamilton Rating Scale for Anxiety, data on mean anxiety score in each arm (active and sham) at baseline and post-treatment were collected and used for meta-analysis. In studies that had a single arm, the data included all patients’ pre- and post-treatment anxiety ratings. With the inclusion of these 2 types of data, the overall data included 2 formats: (1) paired outcome data when studies did not have a comparison arm, and (2) the data in each arm: active treatment and sham. We used Comprehensive Meta-Analysis software (Version 3, developed by Biostat Inc., NJ, USA) to conduct this meta-analysis. The random-effects model was used (Borenstein et al., 2010), which included the point estimate of effect size, standard error, variance, and 95% confidence interval of the point estimate (upper limit and lower limit of the point estimate). Null hypothesis that the treatment with TMS had no greater benefit than sham was tested with 2-tail Z value along with the reported *P* value for the test of null hypothesis. Statistical heterogeneity was quantified with standard measures: the *Q* statistic (i.e., weighted sum of squared differences between individual study effects and the pooled effect across trials), degrees of freedom for *Q* statistic and a *P* value for *Q* statistic, *I*^2^ (i.e., heterogeneity-related variance rather than variance attributed to sampling error), and *τ*^2^ (i.e., variance among true effect sizes).

A total of 6 clinical studies were found after the literature search. Three studies used a 2-arm randomized controlled trial design with active and sham TMS. Three studies were open-label in nature as all participants received active TMS. All studies included results of mean differences in anxiety rating scores before and after the TMS treatment. The 6 studies included in the analysis involved a total of 152 patients and included 97 patients who received active treatment and 55 who received placebo. The studies included delivery of total TMS sessions ranging from 6 to 36. All studies were conducted in the outpatients.

The results are in Figure 1. The meta-analytic estimate of standardized mean difference is −1.857 (confidence interval: −2.219 to −1.494; *P* < .001), indicating that, on average, TMS decreased anxiety scores by −1.857 SDs compared with the sham treatment. There was moderate heterogeneity. Q was 5.768 (*P* = .329) and I^2^ was 13.32%. The variance of true effects, *τ*^2^, was 0.028. Finally, the prediction interval was −2.55 to −1.16, meaning that the true effect size in 95% of all comparable populations falls in this range. The prediction interval range does not include zero and implies consistent effects across all studies in one direction, that is, reducing anxiety scores. We would expect that in some 95% of all populations comparable with those in the analysis, the true effect size will fall in this range. Collectively, the results suggest that TMS treatment for GAD reduced anxiety scores across all populations.

**Figure 1.**
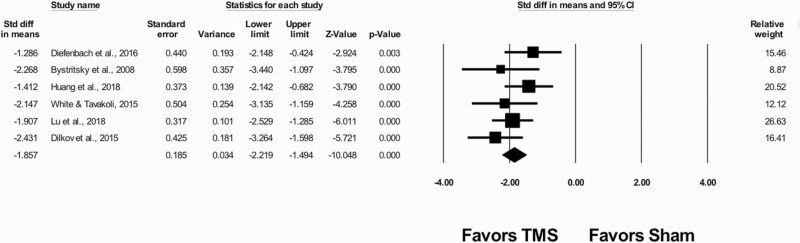
Transcranial magnetic stimulation (TMS) in adults with generalized anxiety disorder.

This review and meta-analysis suggest that TMS has promise as a treatment for adults with GAD and produces large effect sizes similar to or greater than meta-analyses of TMS in major depressive disorder. Further, TMS has been associated with improved emotion regulation in GAD, which may have transdiagnostic application across other anxiety disorders as well as posttraumatic stress disorder and obsessive-compulsive disorder, which can also be characterized by emotional dysregulation (Diefenbach et al., 2016). Last, future studies that incorporate TMS and parallel examinations of the pathophysiology of GAD may reveal novel predictors of treatment response and enhance our understanding of the pathoetiology of GAD. While our findings are not sufficient to change clinical practice, our findings suggest the potential utility of TMS in GAD. A major question would be whether TMS could be considered a first-, second-, or third-line treatment. Overall, safety data are promising. Seizure is one of the most significant overall risks, and it is important to follow appropriate treatment guidelines and available evidence to reduce the change of seizures (Wassermann, 1998; Rossi et al., 2009). Our literature search indicates the overall safety of TMS in those with GAD, and no significant serious adverse events were noted to be attributable to TMS except for 1 case of generalized tonic-clonic seizure in 1 of the 6 studies included in our analysis.

The significant limitations of this systematic review and meta-analysis are (1) only 6 studies were available, each with relatively small sample sizes; (2) open-label and randomized controlled studies were combined for the estimated effect sizes as part of meta-analysis; and (3) studies were conducted in different countries. The following are particular strengths of this article: (1) since the GAD is not currently US Food and Drug Administration approved, a meta-analytic approach could guide researchers in the future and effect size derived from such work has a value in this regard; and (2) we report the detailed biostatistical outcome data previously not presented in the meta-analysis on this topic.

Although there are limitations as outlined in this article, the meta-analysis findings indicate robust response in GAD using TMS. If these can be replicated in satisfactory clinical studies, there is a potential for indicated TMS use in GAD for the adults based on this data. At this time, TMS carries no indication in the adolescent population. In the context of the onset of anxiety disorders being early in life, adolescent use of TMS is particularly worth exploring. TMS represents an emerging treatment that may have significant clinical utility in patients with GAD, a debilitating psychiatric disorder with modest responsiveness to conventional treatment strategies. Currently, however, there are few randomized sham-controlled clinical trials, and further research is warranted, including in the adolescent population.

## Interest Statement

Dr Parikh does not have any conflicts of interest to disclose. Dr Strawn has received research support from the NIH (NIMH/NIEHS/NICHD), Allergan, Otsuka, and Neuronetics. He received material support from and provided consultation to Myriad Genetics/Intracellular Therapeutics and receives royalties from the publication of 2 texts (Springer). Dr Strawn serves as an author for UpToDate, an associate editor for *Current Psychiatry*, and has received honoraria from CMEology, Genomind, and Neuroscience Educational Institute. Dr Walkup has received royalties from Guilford Press and Oxford University Press for multi-author books published about Tourette’s syndrome and from Wolters Kluwer for CME activity on childhood anxiety. He has served as an unpaid advisor to the Anxiety Disorders Association of America and the Trichotillomania Learning Center and an unpaid director on the board of directors of the Tourette Association of American. He has served as a paid speaker for the American Academy of Child and Adolescent Psychiatry, the American Psychiatric Association, and American Academy of Pediatrics. Dr Croarkin has received research grant support from Mayo Foundation for Education and Research, Neuronetics, Inc.; NeoSync, Inc.; NIMH, and Pfizer, Inc. He has received grant-in-kind (equipment support for research studies) from Assurex; MagVenture, Inc; and Neuronetics, Inc. He has served as a paid consultant for Engrail Therapeutics, Myriad Neuroscience, Procter & Gamble, and Sunovion.

## References

[CIT0001] American Psychiatric Association (2013) Diagnostic and statistical manual of mental disorders (5th ed.). Arlington, VA: American Psychiatric Association. doi: 10.1176/appi.books.9780890425596

[CIT0002] Ansara ED (2020) Management of treatment-resistant generalized anxiety disorder. Ment Health Clin 10:326–334. 33224690 10.9740/mhc.2020.11.326 PMC7653736

[CIT0003] Borenstein M , Hedges LV, Higgins JP, Rothstein HR (2010) A basic introduction to fixed-effect and random-effects models for meta-analysis. Res Synth Methods 1:97–111. 26061376 10.1002/jrsm.12

[CIT0004] Diefenbach GJ , Assaf M, Goethe JW, Gueorguieva R, Tolin DF (2016) Improvements in emotion regulation following repetitive transcranial magnetic stimulation for generalized anxiety disorder. J Anxiety Disord 43:1–7. 27467027 10.1016/j.janxdis.2016.07.002

[CIT0005] Lisanby SH , Kinnunen LH, Crupain MJ (2002) Applications of TMS to therapy in psychiatry. J Clin Neurophysiol 19:344–360. 12436089 10.1097/00004691-200208000-00007

[CIT0006] Revicki DA , Travers K, Wyrwich KW, Svedsäter H, Locklear J, Mattera MS, Sheehan DV, Montgomery S (2012) Humanistic and economic burden of generalized anxiety disorder in North America and Europe. J Affect Disord 140:103–112. 22154706 10.1016/j.jad.2011.11.014

[CIT0007] Rossi S , Hallett M, Rossini PM, Pascual-Leone A; Safety of TMS Consensus Group (2009) Safety, ethical considerations, and application guidelines for the use of transcranial magnetic stimulation in clinical practice and research. Clin Neurophysiol 120:2008–2039. 19833552 10.1016/j.clinph.2009.08.016 PMC3260536

[CIT0008] Strawn JR , Lu L, Peris TS, Levine A, Walkup JT (2021) Research review: pediatric anxiety disorders - what have we learnt in the last 10 years? J Child Psychol Psychiatry 62:114–139. 32500537 10.1111/jcpp.13262 PMC7718323

[CIT0009] Wassermann EM (1998) Risk and safety of repetitive transcranial magnetic stimulation: report and suggested guidelines from the International Workshop on the Safety of Repetitive Transcranial Magnetic Stimulation, June 5–7, 1996. Electroencephalogr Clin Neurophysiol 108:1–16. 9474057 10.1016/s0168-5597(97)00096-8

